# Identification of OxyR as an activator of type 1 fimbriae (*fim*) in *Salmonella enterica* serovar Typhi

**DOI:** 10.1128/spectrum.03267-24

**Published:** 2025-09-15

**Authors:** Karine Dufresne, Camille Ou, Melvina Badet, Gabrielle Cadieux, France Daigle

**Affiliations:** 1 Department of Microbiology, Infectiology and Immunology, Université de Montréal 236710 , Montreal, Québec, Canada; 2 CRIPA, Swine and Poultry Infectious Diseases Research Center, Faculty of Veterinary Medicine, University of Montreal 70354, Saint-Hyacinthe, Québec, Canada; Institute of Parasitology, ASCR, Ceske Budejovice, Czech Republic

**Keywords:** type 1 fimbriae, regulator, expression, *S*. Typhi

## Abstract

*Salmonella enterica* serovar Typhi (*S*. Typhi) encodes 14 fimbrial gene clusters, including the mannose-binding type 1 fimbriae known as Fim. Type 1 fimbriae have been implicated in biofilm formation and adhesion to host cells in *Salmonella*. However, their regulation in *S*. Typhi remains largely unknown. To identify genes affecting the regulation of *fim* in *S*. Typhi, we employed both a targeted and a genome-wide transposon-based screening approach. Overall, we identified 18 potential regulators of *fim* expression: 10 activators and 8 repressors. Two genes involved in the electron transport chain, *yqiC* and *ndh,* which encode the type II NADH dehydrogenase NDH-2, were identified. Both YqiC and NDH-2 contribute to the production of reactive oxygen species, prompting an investigation into the roles of oxidative stress response regulators OxyR and SoxR. We found that only OxyR regulates *fim* expression, which was specific to *S*. Typhi. OxyR acts by directly binding to the *fimA* promoter region. This study paves the way for future development of anti-adhesion strategies through the identification of 14 novel regulators for the most prominent fimbriae of *S*. Typhi.

**IMPORTANCE:**

Adhesion mediated by fimbriae is one of the critical steps in the infection process. Therefore, it is essential to better understand the regulation of type 1 fimbriae (*fim*) in the human-specific pathogen *Salmonella enterica* serovar Typhi, the etiologic agent of typhoid fever. In this study, we identified 18 distinct mutants with altered regulation of *fim*. Furthermore, we confirmed that the DNA-binding protein OxyR directly regulates *fim* expression. Importantly, we also demonstrated regulatory differences in *fim* expression between *S*. Typhi and *S*. Typhimurium, as six of the genes identified altering *fim* expression in *S*. Typhi either did not affect *fim* expression in *S*. Typhimurium or had the contrary effect. This highlights fundamental differences between these serovars and emphasizes the need to investigate and compare aspects of gene regulation in *S*. Typhi.

## INTRODUCTION

The *Salmonella enterica* serovar Typhi genome encodes 14 adhesion systems called fimbriae. Among these, the chaperone-usher fimbriae of *Salmonella* represent the most diverse fimbrial class, comprising 12 systems, including the type 1 fimbriae (T1F), named Fim (1). T1F are among the most extensively studied adhesion systems and are the most common fimbriae within the *Enterobacteriaceae* family (2–4). However, their regulation and function are distinct (5–7). *Escherichia coli* T1F, encoded by the *fim* operon, is well studied and is regulated by a mechanism known as phase variation, which switches the operon promoter between an OFF state and an ON state (8). This phase variation is controlled by multiple regulators (9). In *Salmonella,* the *fim* operon is accompanied and regulated by ancillary genes (*fimZ*, *fimY*, *fimW*, *STM0551,* and *fimU*) (10–12). Among these regulators, FimZ is the major activator of the *Salmonella fim* operon. Its activity is modulated by other ancillary proteins: FimY enhances activation, whereas FimW acts as a repressor. Beyond the ancillary genes, the expression of the *fim* operon is influenced by a range of other factors, including Lrp, YqiC, and IprA (YaiV) in *S*. Typhimurium (3, 13–15). Interestingly, *Salmonella* T1F are the only fimbriae expressed under laboratory conditions (16). Structurally, *Salmonella* T1F are composed of repeated units of FimA, the major subunit, along with minor subunits such as FimF and the adhesin FimH (17, 18). The FimH specifically binds to high-mannose oligosaccharides on eukaryotic extracellular glycoproteins (3). Variants of FimH have been observed across various serovars, leading to differences in adhesion to host cells, virulence in mice, and yeast agglutination (19, 20). These FimH variants differ by only a few amino acids; however, they exhibit significant differences in binding phenotypes (20).

A delicate balance in the expression of virulence factors allows *Salmonella* to effectively establish infection. This equilibrium is maintained by various regulators that modulate the expression of T1F and other virulence factors. In the intestinal tract, bacterial cells are exposed to numerous environmental stresses, such as nutrient limitation, acidic pH environment, thermal stress, reactive oxygen species (ROS), and osmotic stress. For an enteric pathogen such as *Salmonella*, the ability to regulate its responses to these environmental stresses is crucial for effective virulence (3).

In this study, we aimed to identify regulators of *fim* expression in *S*. Typhi. We tested various candidate regulators and screened a library of mutants generated by transposon insertion. We identified 18 mutants as factors modulating T1F expression. Additionally, we confirmed the direct role of OxyR as an activator of *fim* expression, a function specific to *S*. Typhi.

## MATERIALS AND METHODS

### Bacteria, plasmids, and growth conditions

Strains and plasmids are listed in Table S1. Bacteria were cultured on Luria-Bertani (LB) agar or LB broth. Experiments were routinely performed in LB broth except if cited otherwise. Antibiotics or supplements were used at the following concentrations: 34 µg/mL chloramphenicol, 50 µg/mL kanamycin, 100 µg/mL ampicillin, and 50 µg/mL diaminopimelic acid. Introduction of plasmid in specific strains was performed by electroporation (21).

### Cloning of *fimA* promoter and chromosomal deletion of putative regulator genes

The fusion of the *fimA* promoter to the *lacZ* gene on the pRS415 vector (pRS*fimA*) was described in reference 22. Mutant strains of putative regulators were constructed by allelic exchange mutagenesis as described in reference 23. Non-polar deletions were confirmed by PCR and Sanger sequencing (data not shown).

### Screening of transposon-based library on MacConkey agar

The vector pRS*fimA* Cm (pSIF519) was transformed into a transposon-based mutant library (Tn*10*-based library) constructed by Sabbagh et al. (24). The library was screened on MacConkey agar (Dibco) for the expression of the *fimA* promoter. The wild-type strain harboring the vector without (pRS415) or with *fim* promoter region (pRS*fimA*) was incubated simultaneously on MacConkey as negative and positive controls, respectively. For each mutant isolated, the 3′ region of the transposon was amplified by nested PCR using semi-random primers (25). The PCR products were sequenced (IRIC) and identified with BLAST (NCBI).

### β-galactosidase assays

The expression of the *fimA* promoter was assessed by a β-galactosidase assay by adding the *fimA-lacZ* plasmid (pSIF474) in the different strains or the plasmid *fimA-lacZ* Cm (pSIF519) in the transposon library, as described in reference 26. Cells were grown in LB broth overnight at 37°C with agitation, unless indicated. After cell lysis, β-galactosidase assay was performed using *o*-nitrophenyl-β-D-galactopyranoside. Optical density at 420 and 550 nm was monitored, and Miller units were calculated. To validate the significance of the difference in *fimA* expression, an unpaired *t*-test was performed between the wild-type and mutant strains, and a *P* value of <0.005 was considered significant.

### Peroxide sensitivity

Sensitivity to hydrogen peroxide was assessed using agar diffusion assays on LB solid media. Growth inhibition was measured by mixing 0.1 mL of an overnight culture grown in LB medium with 3 mL of molten top agar (maintained at 50°C), which was then poured onto an LB plate. A filter disc (10 mm, Whatman No. 1) soaked in 10 µL of 30% H_₂_O_₂_ solution was placed at the center of the solidified top agar. After 18 h of incubation, the diameter of the clear zone of inhibition was measured.

### 6xHis-OxyR production and purification

OxyR of ISP1820 was cloned into pET14b by PCR using OxyR_F_NdeI/OxyR_R_BamHI primers, NdeI/BamHI restriction enzyme (Anza), and ligase T4 (NEB). The vector was then transformed into *E. coli* BL21 (DE3). 6xHis-OxyR production and cell harvest were performed following the pET system protocol (Novagen). A concentration of 0.1 mM IPTG was used for induction, and the cells were incubated overnight at 25°C. After cell lysis by sonication, the soluble fraction was isolated to harvest 6xHis-Oxy, followed by protein purification using gravity affinity chromatography (Bio-Rad) with NTA resin (Thermo-Fisher).

### Electrophoretic mobility shift assay

Electrophoretic mobility shift assay (EMSA) was performed using purified 6xHis-OxyR with *pfim*, *pkatG* and *recA* as DNA probes. The probes were amplified by PCR using FimA prom F/FimA prom R2, KatG F1/KatG R2, and RecA F1/RecA R2 pairs of primers, respectively. A 10 nM concentration of probe was used in all mixtures with different concentrations of proteins. EMSA was carried out as described previously (27). After migration, the gel was stained with 1,000× RedSafe Nucleic Acid Staining Dye in migration buffer for 10 minutes, washed with ddH_2_O, and imaged using Proteinsimple AlphaImager HP device and software.

### FimA production

The wild-type, *fimA* deletion mutant, *oxyR* deletion mutant, and the *oxyR*-complemented strains were incubated overnight with agitation at 37°C in LB. Bacteria were thermally shocked (60°C) and then vortexed. Supernatants were collected to a volume equivalent to 100 absorbance units (OD_600_) to normalize samples for analysis. The extracellular proteins were precipitated with 10% TCA (vol/vol) on ice for 30 minutes. The TCA-insoluble fraction was collected by centrifugation, washed with ice-cold acetone, and resuspended in an appropriate volume of SDS-PAGE loading buffer (62.5 mM Tris/HCl, pH 6.8, 10% [vol/vol] glycerol, 2% [wt/vol] SDS, 0.05% [wt/vol] *β*-mercaptoethanol, and 0.05 [wt/vol] bromophenol blue). Proteins were separated on Any Kd precast gels (Bio-Rad) and stained with Ready Blue (Sigma) or transferred onto PVDF membrane. The membrane was blocked overnight in Tris-buffered saline containing 0.1% (vol/vol) Tween 20 (TBST) and 5% (wt/vol) non-fat dried milk at 4°C. Blots were then incubated for 1 h at room temperature with rabbit affinity-purified antibodies raised against FimA (1: 100) (kindly provided by A. J. Bäumler) in TBST with 2.5% (wt/vol) non-fat dried milk. Peroxidase-conjugated AffiniPure goat anti-rabbit IgG (Jackson ImmunoResearch Laboratories) was used as the secondary antibody (1:10,000) in TBST with 2.5% (wt/vol) non-fat dried milk for 1 h at room temperature. Immobilon Western (Millipore) was used as the chemiluminescent substrate for the detection of the antibody complexes. Blot images were acquired with a Fusion FX7 (Montreal Biotech Inc.).

## RESULTS

### Identification of type 1 fimbriae regulators by targeted and transposon-based library screening

**Fig 1:**
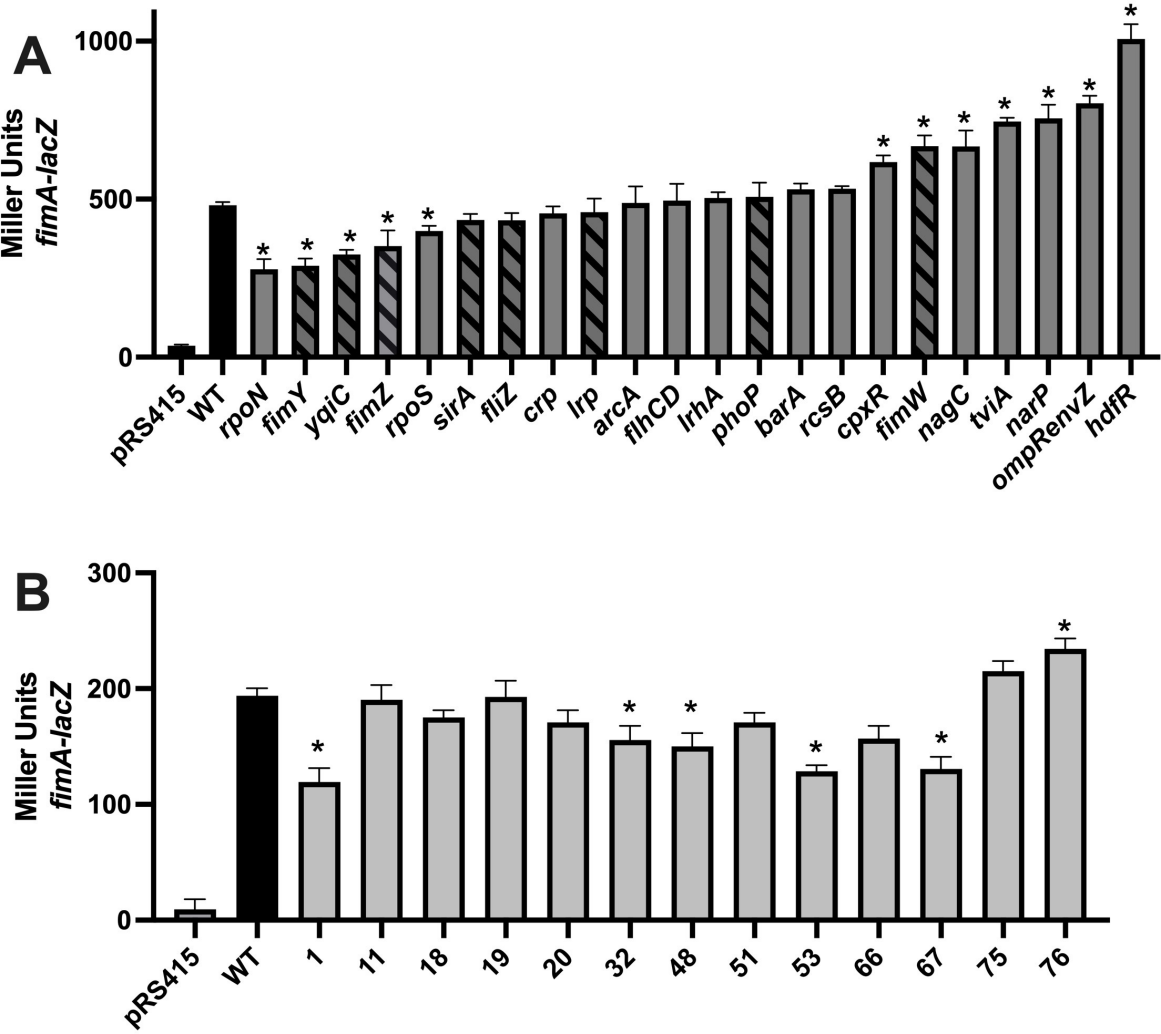
Expression of *fimA* in global regulators and transposon mutants. Expression of *fimA* was evaluated by β-galactosidase assay. (**A**) Candidate regulators containing the *fimA-lacZ* reporter (pSIF474) were compared to the wild-type (WT) strain, with striped bars indicating genes previously known to be involved in *fim* regulation in *S*. Typhimurium. (**B**) *fimA-lacZ* reporter (pSIF519) expression in transposon mutants identified from the transposon library screening. Results are presented as the mean Miller units ± SEM from duplicate assays of biological triplicates. Mutants with a significant difference (*P* < 0.005) compared to WT in the β-galactosidase assay are marked with an asterisk.

To identify *fim* regulators in *S*. Typhi, we tested known regulators from the closely related *S*. Typhimurium, as well as several global regulators using *lacZ* reporter gene (pSIF474). Twenty-two selected isogenic mutants were constructed, and *fimA* expression was evaluated (Fig. 1A). The deletion of *rpoN*, *fimY*, *yqiC, fimZ,* and *rpoS* resulted in significantly lower *fimA* expression compared to the wild type, suggesting their potential roles as activators of *fimA* expression. In contrast, the deletion of *cpxR*, *fimW*, *nagC*, *tviA*, *narP*, *ompR/envZ,* and *hdfR* led to increased *fimA* expression, indicating their roles as potential repressors. The deletion of *sirA, fliZ, crp, lrp, arcA, flhCD, lrhA, phoP, barA,* and *rcsB* had no significant impact on *fimA* expression. Among the eight regulators known to modulate *fim* in *S*. Typhimurium, only *fimW, fimY*, *fimZ,* and *yqiC* were also found to regulate *fimA* in *S*. Typhi.

To identify novel regulators of T1F in *S*. Typhi, the *fimA-lacZ* expression vector was transformed into a Tn*10*-based library of *S*. Typhi (24), and the resulting colonies were screened on MacConkey agar. Colonies exhibiting a LacZ phenotype altered from the wild-type were selected, and their expression was confirmed by β-galactosidase assay (Fig. 1B). Six candidates were confirmed to have significantly different levels of *fimA* expression, and the locations of the transposon insertions were identified by Sanger sequencing (Table 1).

**TABLE 1:** Identification of transposon insertions as significant modulators of *fim*

Screening name	Gene with transposon insertion	Function
1	*ndh*	Type 2 NADH dehydrogenase (NDH-2)
32	STY4579	Putative membrane protein
48	*yeeF*	Putative transmembrane amino acid transporter
53	STY1861 (*yddO*)	ATP-binding cassette domain-containing protein
67	*celD*	Putative *cel* operon repressor
76	*waaK (rfaK*)	Lipopolysaccharide 1,2-N-acetylglucosaminetransferase

### Role of the electron transport chain in *fim* regulation

Two different factors involved in the electron transport chain (ETC), YqiC and NDH-2 (*ndh*), were identified as activators of T1F expression. YqiC is a multifunctional protein that interacts with subunits of Complex II (28) and acts as an accessory factor for ubiquinone, which is essential for the electron transport (29, 30). NDH-2 (*ndh*) is a NADH dehydrogenase that accepts and transfers electrons from Complex I to the quinone pool between Complexes II and III. It plays a major role in aerobic respiration in bacteria by regulating the balance between NADH and NAD (31–33). Given that Complex I in the ETC involves a type I NADH dehydrogenase, we aimed to determine the role of ETC Complex I in T1F expression.

**Fig 2:**
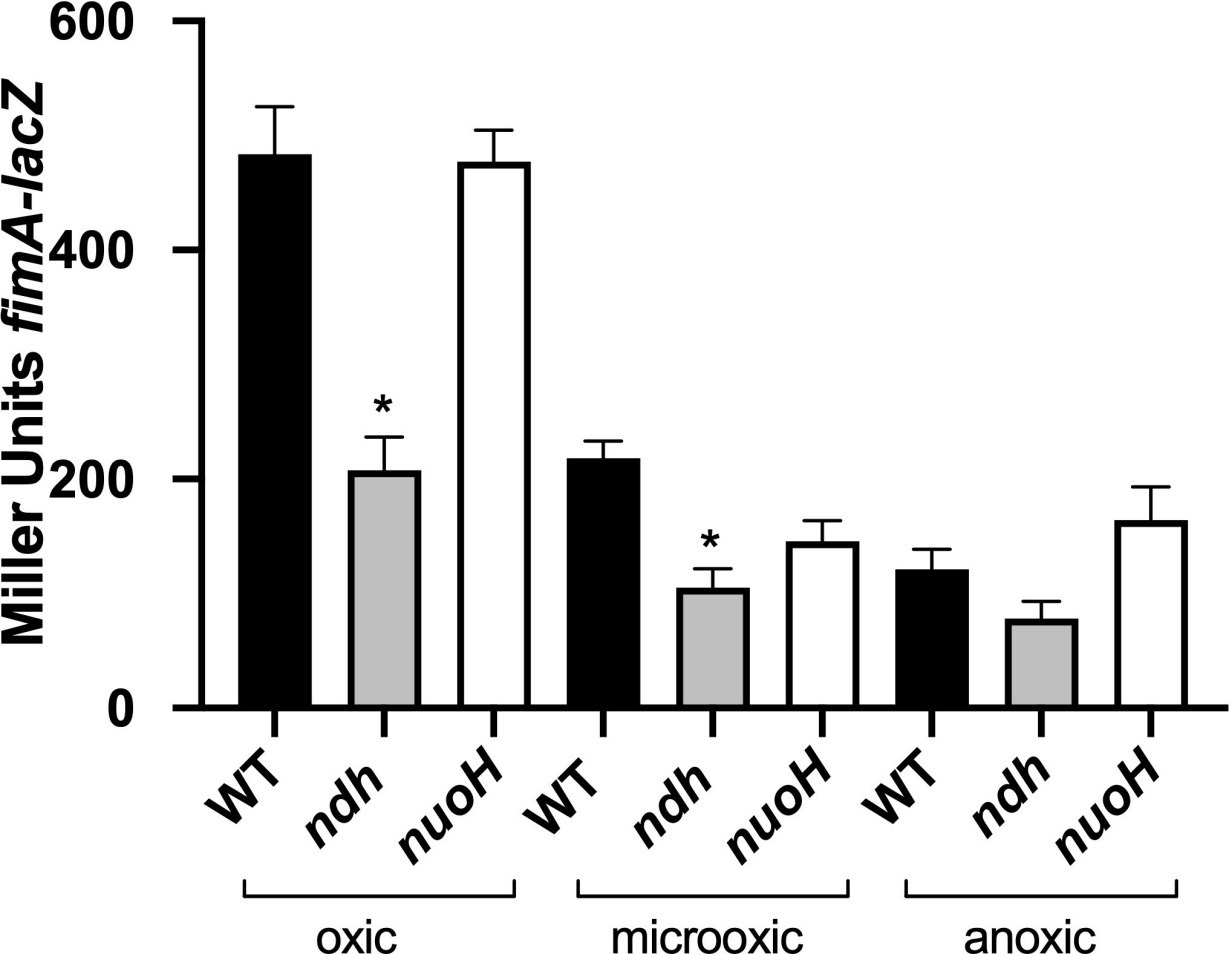
Role of NADH dehydrogenase and the effect of oxygen. Expression of *fimA* was compared between the mutants and the wild type under different oxygen conditions: shaking (oxic), standing (microoxic), and in an anaerobic chamber (anoxic). Results are presented as the mean Miller units ± SEM from duplicate assays of biological triplicates. Mutants with a significant difference (*P* < 0.005) compared to WT in the β-galactosidase assay are marked with an asterisk.

We investigated whether changes in T1F expression were associated with the loss of the enzymatic activity by evaluating *fimA* expression in mutants of NADH dehydrogenase 1, NDH-1 (*nuoH*) and NDH-2 (*ndh*). Since NDH-2 is primarily used during aerobic respiration (34) and repressed under anoxic conditions (35), we assessed *fimA* expression in the NDH-1 and NDH-2 mutants under varying oxygen levels (oxic, microoxic, and anoxic). *fimA* expression was significantly different in the *ndh* mutant under oxic and microoxic conditions, while the *nuoH* mutant showed no significant difference from the wild type under any tested conditions (Fig. 2). This suggests that NDH-1 does not play a role in the regulation of *fim* fimbriae, and NDH-2 is important for the increase of *fim* expression in the presence of oxygen only.

### Role of oxidative stress on T1F expression

**Fig 3:**
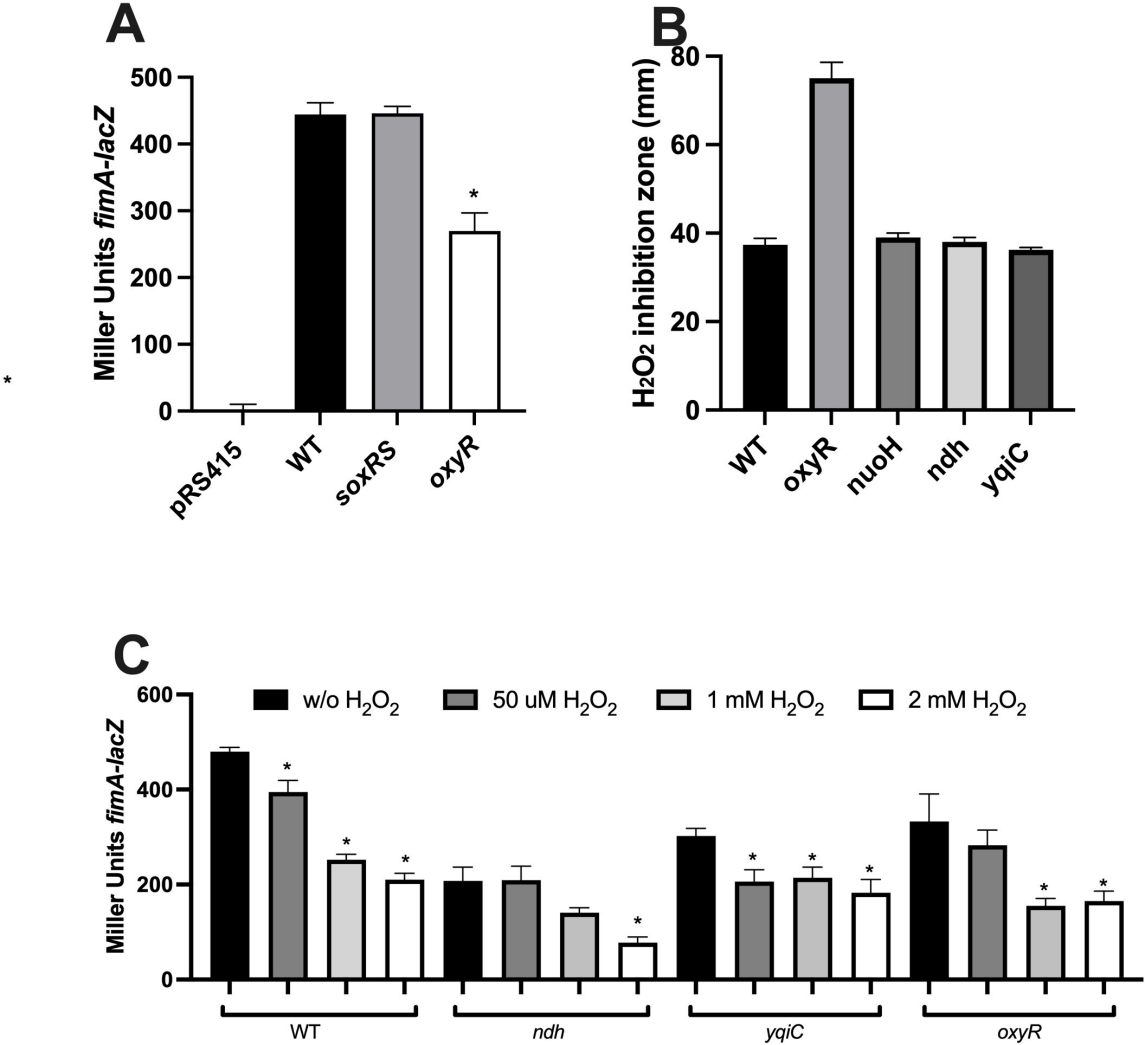
Role of oxidative stress on *fimA* expression. (**A**) Expression of *fimA* was evaluated in oxidative regulator mutants *soxRS* and *oxyR*. (**B**) Determination of hydrogen peroxide sensitivity using the disk diffusion assay. Growth inhibition was measured (in millimeters) after 18 h for different mutants and compared with the wild type. (**C**) Exposure to different concentrations (subminimal) of hydrogen peroxide on *fimA* expression in different mutants. Mutants with a significant difference (*P* < 0.005) compared to WT are marked with an asterisk. Results are presented as the mean from biological triplicates.

As *yqiC* has been shown to induce oxidative stress response in *Escherichia coli* (14), and because, unlike NDH-1, NDH-2 can generate reactive oxygen species, such as superoxide and hydrogen peroxide (36), we investigated the role of oxidative stress on *fimA* expression. Specifically, we examined the roles of oxidative stress transcriptional regulators OxyR and SoxR, which respond to hydrogen peroxide and superoxide, respectively, by activating the expression of catalases and peroxidases to degrade ROS. Interestingly, a significant difference in *fimA* expression was observed only in the *oxyR* mutant (Fig. 3A). This suggests that *fim* expression may be linked to oxidative stress induced specifically by hydrogen peroxide.

We evaluated the sensitivity to hydrogen peroxide using the disk diffusion assay in the *oxyR* and ETC mutants. Among these, only the *oxyR* mutant demonstrated greater sensitivity compared to the wild type (Fig. 3B). Subsequently, we assessed *fimA* expression in the wild type, *ndh, yqiC,* and *oxyR* mutants during growth in the presence of increasing concentrations of hydrogen peroxide (Fig. 3C). The expression of *fimA* decreased proportionally with increasing peroxide concentrations and was significantly reduced at 2 mM in all mutants.

### OxyR regulates the *S*. Typhi *fim* operon

**Fig 4:**
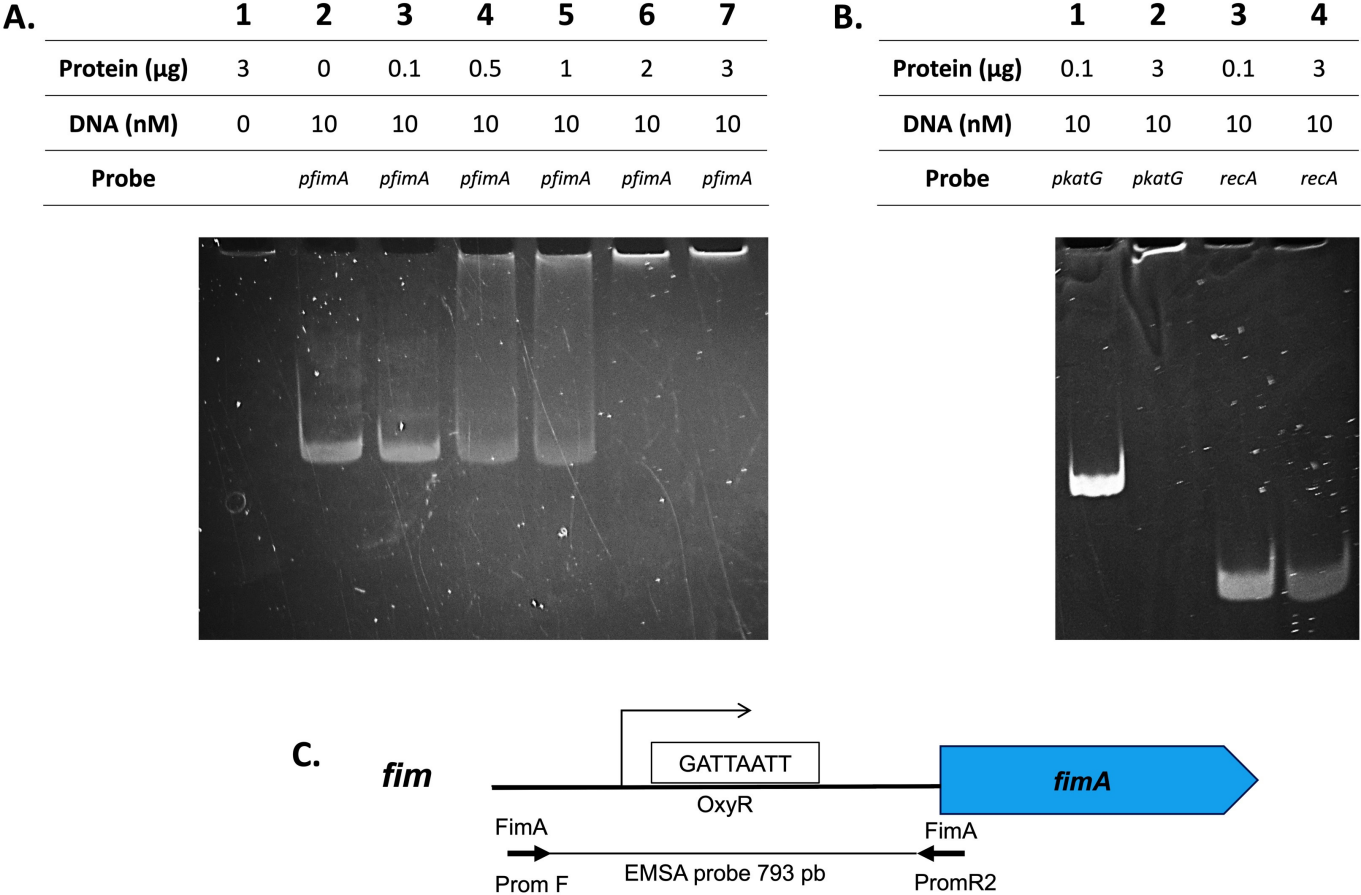
OxyR binding site on *fim* promoter region. EMSA was performed using 6xHis-OxyR protein and DNA probes on a 5% acrylamide gel stained with RedSafe Nucleic Acid Staining Solution. (**A**) The lanes are as follows: (1) control well containing only 6xHis-OxyR protein. (2) Control well containing only the DNA probe. (3–7) Increasing concentrations of the 6xHis-OxyR protein, ranging from 0.1 to 3 µg on *pfimA* (*fim* promoter) probe. (**B**) 6xHis-OxyR protein at concentrations of 0.1 µg (lane 1) and 3 µg (lane 2) incubated with *pkatG* (*katG* promoter) probe, used as a positive control for protein-DNA binding. The same concentrations of 6xHis-OxyR protein were used for the assay with the *recA* probe, used as a negative control for protein-DNA binding. (**C**) Genetic organization of the *fim* operon with the putative OxyR binding site and the probe fragment used for the EMSA.

OxyR is a regulatory protein that binds to the promoters of its regulated genes, including the catalase gene *katG* (37). An OxyR binding site sequence was identified *in silico* (Using Bprom, Softberry) within the *fim* promoter region of *S*. Typhi. To investigate whether OxyR directly regulates the *fimA* gene, DNA corresponding to the promoter regions of *fimA, katG,* and part of the *recA* gene at 3′ was analyzed using electrophoretic mobility shift assays with purified 6xHis-OxyR protein (Fig. 4). OxyR successfully bound the *katG* promoter, confirming the functionality of the purified OxyR protein, and did not bind to *recA.* For p*fimA,* partial binding was observed with 0.5 µg of protein, while a complete binding shift occurred with 2 µg of protein. These findings suggest that OxyR directly regulates *fim* expression.

### FimA production

**Fig 5:**
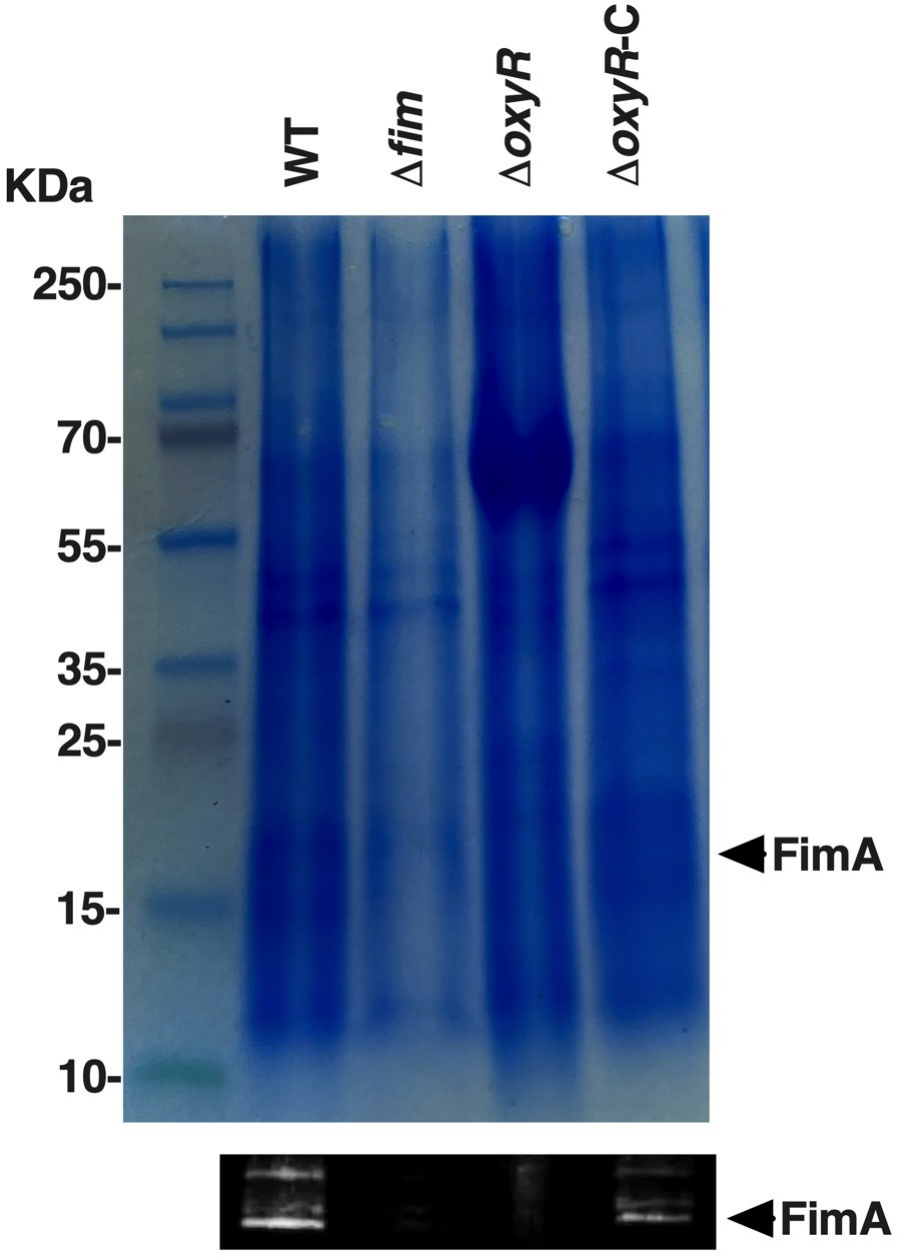
FimA is undetectable in ISP1820 *ΔoxyR*. Detection of FimA by Western blot was performed on *S*. Typhi wild type, *fim* mutant, *oxyR* mutant, and the complemented *oxyR* mutant strains. Supernatants were normalized at 100 absorbance units (OD_600_) before TCA precipitation (10%, vol/vol). Representative SDS-PAGE and anti-FimA Western Blot are shown. *S*. Typhi FimA expected size = 18.793 kDa.

To validate the influence of OxyR on Fim production, we evaluate the expression of FimA after a fimbrial extraction in different background strains by Western blot (Fig. 5). FimA was detected only in the wild-type strain or the *oxyR* complemented strain, confirming that OxyR acts as an activator of the Fim operon.

### Regulation of other *S*. Typhi fimbriae by OxyR

**Fig 6:**
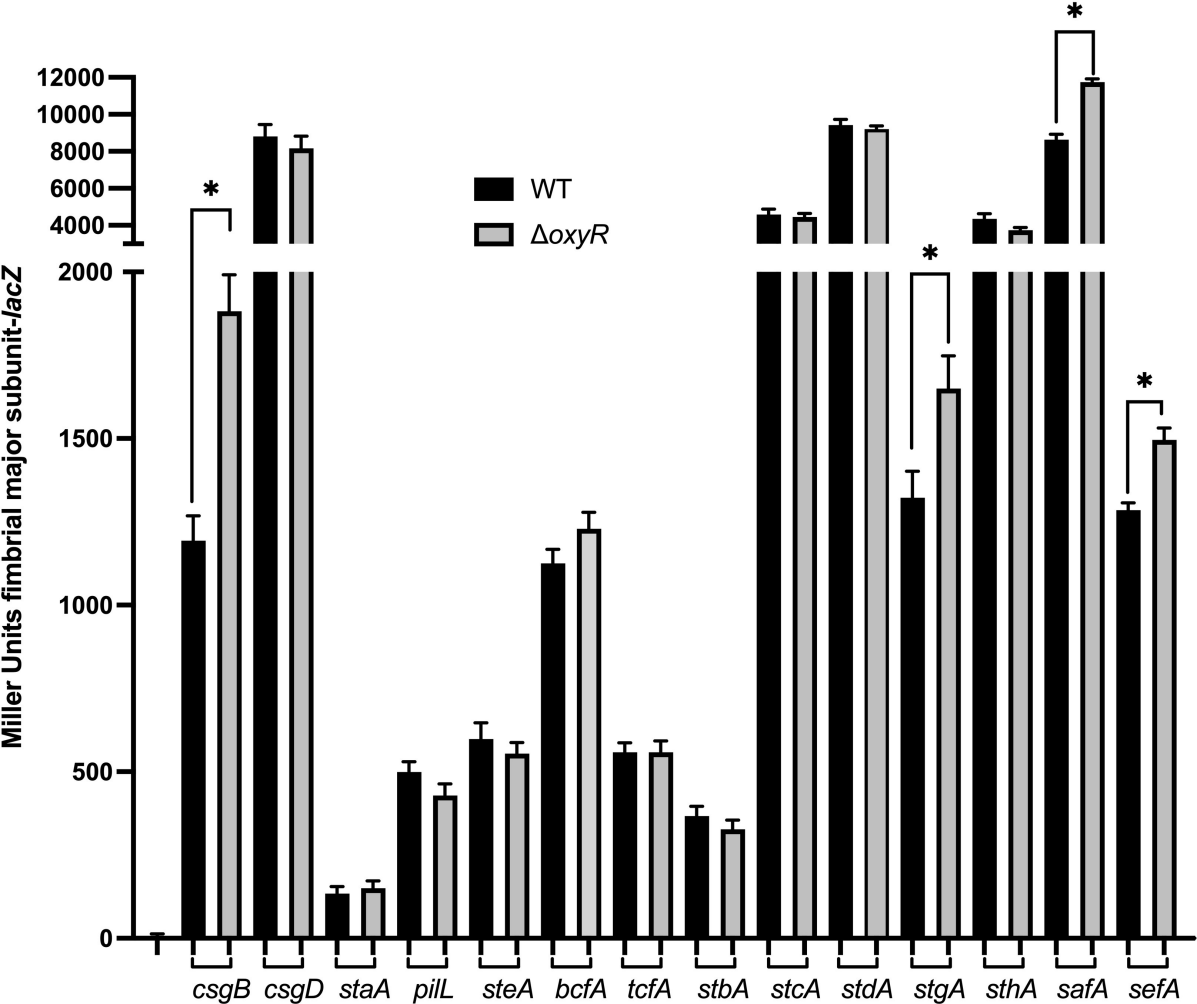
Role of OxyR in different fimbrial systems of *S*. Typhi. Fimbrial expression was compared between the wild type (WT) and the *oxyR* mutant. Results are presented as the mean Miller units ± SEM from duplicate assays of biological triplicates. Mutants with a significant difference (*P* < 0.005) compared to WT in the β-galactosidase assay are marked with an asterisk.

The *S*. Typhi genome contains 14 fimbrial gene clusters (22, 38). To assess whether the *oxyR* mutation affects the expression of other fimbriae in *S*. Typhi, we compared the expression levels of these fimbriae between the wild-type and the *oxyR* mutant strains. Notably, expression levels of *csgB*, *stgA*, *safA,* and *sefA* were significantly higher in the *oxyR* mutant, suggesting that OxyR acts as a repressor in regulating these fimbrial systems (Fig. 6). In contrast, OxyR acts as an activator of Fim.

### Role of OxyR in regulating *fimA* in *S*. Typhimurium

**Fig 7:**
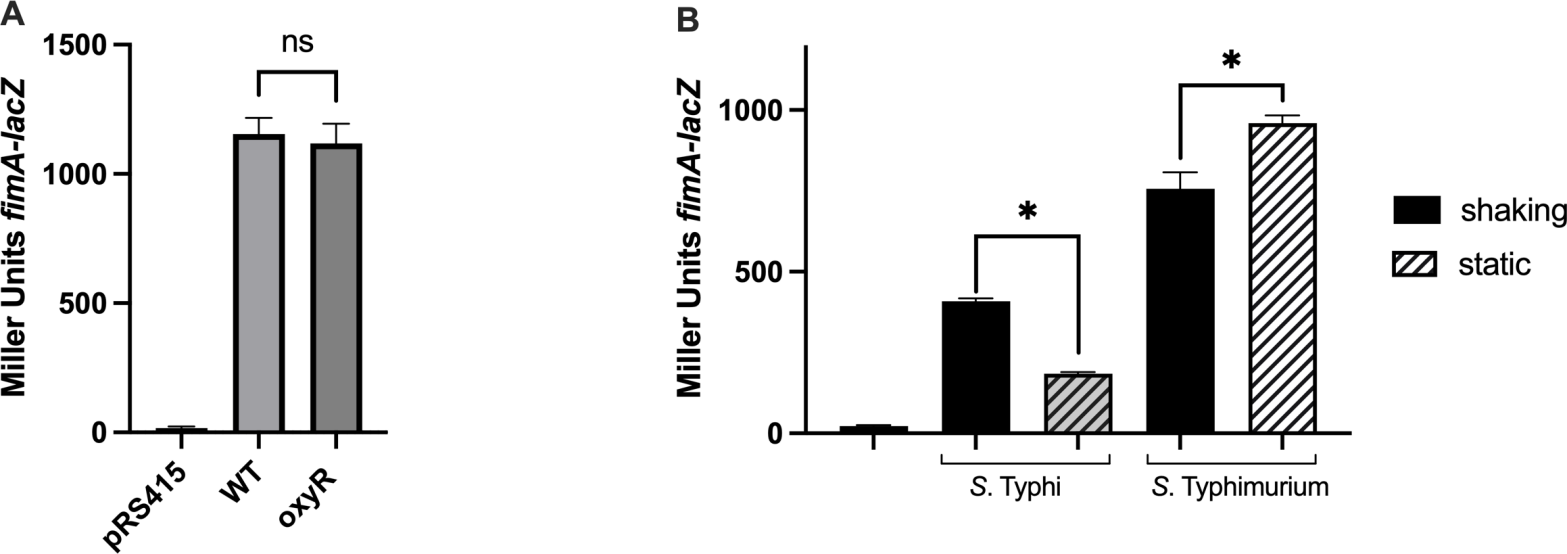
T1F expression in *S*. Typhimurium. (**A**) Expression of *fimA* was evaluated in the wild type (WT) and the oxidative regulator mutant *oxyR* of serovar Typhimurium strain SL1344. (**B**) Expression of *fimA* was evaluated under oxic (shaking) (black) or microoxic (static) (striped) conditions in serovar Typhi and serovar Typhimurium. Significant differences (*P* < 0.005) compared to WT are marked with an asterisk. Results are presented as the mean of biological triplicates.

In this study, we identified that OxyR directly regulates *fimA* expression in *S*. Typhi. To further investigate, we evaluated the role of OxyR on *fimA* expression in the closely related serovar *S*. Typhimurium. Surprisingly, no difference in *fimA* expression was observed between the wild type and the *oxyR* mutant in *S*. Typhimurium, suggesting differences in regulatory mechanisms between these two serovars (Fig. 7A). Since T1F gene expression in *S*. Typhimurium is favored in static liquid medium (39, 40), we then compared *fimA* expression during growth under static and shaking conditions in both *S*. Typhi and *S*. Typhimurium (Fig. 7B). Interestingly, *fimA* expression was lower in *S*. Typhi compared to *S*. Typhimurium, with an inverted pattern also observed: *S*. Typhi showed higher expression under oxic conditions, whereas *S*. Typhimurium exhibited higher expression under microoxic conditions.

## DISCUSSION

To identify factors influencing type 1 fimbriae expression in *S*. Typhi, we analyzed the promoter activity of *fimA* in various regulator mutant strains and screened random mutants from a transposon library. A total of 18 regulators of *fimA* expression were identified, including 14 novel ones. We confirmed that FimY and FimZ act as activators of the *fimA* operon, while FimW inhibits *fimA* expression, consistent with observations previously reported in *S*. Typhimurium (10, 11, 41, 42). However, SirA, FliZ, PhoP, and Lrp did not significantly affect *fimA* expression in *S*. Typhi. This discrepancy may result from differences in the nutritional and environmental conditions used (13). Interestingly, YqiC functions as an activator in *S*. Typhi, in contrast to its role in *S*. Typhimurium, where it acts as an inhibitor of *fimZ* and *fim* operon, as it was shown that *fimA* was upregulated in a *yqiC* mutant (14). Several transcriptional factors were identified, such as NagC, CpxR, TviA, NarP, OmpR, and HdfR, which act as inhibitors, and RpoN, RpoS, and CelD, which serve as activators of *fimA* expression. Globally, perturbations in the bacterial envelope, capsule synthesis, LPS modification, membrane proteins, and extracellular components may modulate *fim* expression. We hypothesize that in the case of envelope perturbations, bacteria prioritize maintaining cellular integrity. Since the synthesis of fimbriae could further destabilize the envelope, the bacteria may suppress fimbrial synthesis to reduce instability. Similarly, TviA, the regulator of the Vi capsule mainly associated with typhoidal serovars, was identified as a repressor of *fimA* expression.

Two *fimA* activators, YqiC and NDH-2 (*ndh*), an ubiquinone synthesis accessory factor and the NADH dehydrogenase-2, respectively, are both involved in the ETC and redox balance. NDH-2 is one of the first electron donors of the ETC in aerobic conditions and controls the balance between NADH and NAD+ (43, 44). Moreover, both YqiC and NDH-2 contribute to the production of reactive oxygen species (13, 14). These proteins are unlikely to be direct regulators of *fimA* but likely affect other regulators that sense imbalances in the ETC or in ROS levels.

By investigating the role of ROS through the oxidative stress regulators OxyR and SoxRS, we identified that only OxyR modulated *fimA* expression (Fig. 3A). OxyR is a key regulator that protects bacteria from oxidative damage by sensing hydrogen peroxide and activating genes involved in detoxification, repair, and redox balance. We showed that the presence of hydrogen peroxide influenced *fimA* expression, independently of the concentration or the genetic background (Fig. 3C). This suggests that the redox balance is more important for *fim* expression than the presence of ROS. We demonstrated that OxyR is directly involved in the regulation of *fimA* by binding to its promoter. Interestingly, we also showed that OxyR modulates the expression of four other fimbriae, *csgB, stgA, safA,* and *sefA* in *S*. Typhi. However, in these systems, OxyR acts as a repressor, in contrast to an activator for *fimA* expression. The ability of OxyR to function as both an activator and a repressor is well established in *E. coli* and depends on several factors, including the location of its binding site relative to the promoter, conformational change upon oxidation, and the presence of co-regulators or other transcriptional regulators (45–47).

In addition to its role in oxidative stress, OxyR has also been implicated in fimbriae and adhesin regulation in various bacterial species, including *E. coli*, *Serratia marcescens, Porphyromonas gingivalis*, *Aggregatibacter actinomycetemcomitans,* and *Klebsiella pneumoniae* (48–52). Interestingly, OxyR can also act as a repressor of these systems (50, 53, 54). Moreover, we showed that OxyR was not involved in *fimA* expression in *Salmonella* serovar Typhimurium (Fig. 6), highlighting another serovar-specific regulatory difference.

Interestingly, both *Salmonella* serovars encounter several stresses during infection, including ROS within host macrophages. However, after ingestion and upon reaching the intestinal barrier, only *S*. Typhi produces the Vi polysaccharide capsule encoded by the *tviB-E* and *vexA-E* genes (55). The Vi capsule acts as a shield against the host’s innate immune defenses by preventing complement deposition and phagocytosis by neutrophils, as well as reducing the inflammatory response (56–58). The Vi capsule is regulated by TviA, OmpR, and RcsB (59–61). In this study, we identified that TviA, the regulator of the Vi capsule, acts as a repressor of *fimA* expression (Fig. 1). Interestingly, TviA is also known to be regulated by OxyR (62, 63). Thus, the Vi capsule, a unique virulence factor of *S*. Typhi, may account for some of the discrepancies observed between these *Salmonella* serovars.

In conclusion, we identified several potential regulators involved in *fim* regulation. Moreover, we established that envelope perturbations repressed *fim* expression in *S*. Typhi. We showed that members of the ETC, YqiC, and NDH-2 are involved in *fim* activation by ROS production and that oxidative stress regulator OxyR directly binds to *fimA* promoter. A better understanding of *fim* regulation will definitively allow a better comprehension of *S*. Typhi pathogenesis, as Fim may represent important fimbriae of *S*. Typhi.

## SUPPLEMENTAL MATERIAL

Table S1 spectrum.03267-24-s0001.docx Table of strains and plasmids.

Table S2 spectrum.03267-24-s0002.docx Table of primers.

ASM does not own the copyrights to Supplemental Material that may be linked to, or accessed through, an article. The authors have granted ASM a non-exclusive, world-wide license to publish the Supplemental Material files. Please contact the corresponding author directly for reuse.
