# Supplementary material for: Identification of OxyR as an activator of type 1 fimbriae (fim) in Salmonella enterica serovar Typhi
Source: Microbiol Spectr. 2025 Sep 15;13(10):e03267-24. doi: 10.1128/spectrum.03267-24 (PMC12502779; doi:10.1128/spectrum.03267-24)
Supplement: Table S1 — Table of strains and plasmids. [file spectrum.03267-24-s0001.docx]

| **Table S1. Bacterial strains and plasmids used for this study** | | | |
| --- | --- | --- | --- |
| **Reference Number** | **Name** | **Characteristic** | **Source or reference** |
| ***S.* Typhi** |  |  |  |
| DEF1045 | WT | ISP1820 wild-type | R. Curtiss III, U. Florida |
| DEF1153 | WT pRS415 | ISP1820 (pRS415) | (Dufresne et al, 2018) |
| DEF1095 | WT pRS*fimA* | ISP1820 (pSIF474) | (Dufresne et al, 2018) |
| DEF1397 | WT pRS Cm | ISP1820 (pRS415 Cm) | This study |
| DEF1398 | WT pRS*fimA* Cm | ISP1820 (pSIF519) | This study |
| DEF1239 | Δ *arcA* | ISP1820 Δ*arcA* | (Murret-Labarthe et al, 2019) |
| DEF569 | Δ *barA* | ISP1820 Δ*barA* | (Murret-Labarthe et al, 2019) |
| DEF1238 | Δ *cpxR* | ISP1820 Δ*cpxR* | (Murret-Labarthe et al, 2019) |
| DEF1342 | Δ *crp* | ISP1820 Δ*crp* | (Dufresne et al, 2020) |
| DEF1338 | Δ *fimW* | ISP1820 Δ*fimW* | This study |
| DEF1316 | Δ *fimY* | ISP1820 Δ*fimY* | This study |
| DEF1309 | Δ *fimZ* | ISP1820 Δf*imZ* | This study |
| DEF571 | Δ *flhCD* | ISP1820 Δ*flhCD* | Sabbagh 2012 |
| DEF1330 | Δ *fliZ* | ISP1820 Δ*fliZ* | This study |
| DEF1355 | Δ *hdfR* | ISP1820 Δ*hdfR* | (Dufresne et al, 2020) |
| DEF432 | Δ *lrhA* | ISP1820 Δ*lrhA* | (Dufresne et al, 2020) |
| DEF1329 | Δ *lrp* | ISP1820 Δ*lrp* | (Dufresne et al, 2020) |
| DEF1336 | Δ *nagC* | ISP1820 Δ*nagC* | (Dufresne et al, 2020) |
| DEF1290 | Δ *narP* | ISP1820 Δ*narP* | (Murret-Labarthe et al, 2019) |
| DEF863 | Δ *ompR/envZ* | ISP1820 Δ*ompR*Δ*envZ* | (Murret-Labarthe et al, 2019) |
| DEF1526 | Δ *oxyR* | ISP1820 Δ*oxyR* | (Dufresne et al, 2020) |
| DEF1241 | Δ *phoP* | ISP1820 Δ*phoP* | (Murret-Labarthe et al, 2019) |
| DEF1319 | Δ *rcsB* | ISP1820 Δ*rcsB* | (Murret-Labarthe et al, 2019) |
| DEF1512 | Δ *rpoN* | ISP1820 Δ*rpoN* | (Dufresne et al, 2020) |
| DEF1769 | Δ *rpoS* | ISP1820 Δ*rpoS* | This study |
| DEF1247 | Δ *sirA* | ISP1820 Δ*sirA* | (Murret-Labarthe et al, 2019) |
| DEF884 | Δ *soxRS* | ISP1820 Δ*soxRS* | (Dufresne et al, 2020) |
| DEF434 | Δ *tviA* | ISP1820 Δ*tviA* | (Dufresne et al, 2020) |
| DEF1328 | *Δ yqiC* | ISP1820 *Δ yqiC* | This study |
| TRASH44 | FIM-1 | ISP1820 *ndh*::Tn10 (pSIF519) | This study |
| TRASH54 | FIM-32 | ISP1820 STY4579::Tn10 (pSIF519) | This study |
| TRASH55 | FIM-48 | ISP1820 *yeeF*::Tn10 (pSIF519) | This study |
| TRASH58 | FIM-53 | ISP1820 *yddO*::Tn10 (pSIF519) | This study |
| TRASH60 | FIM-67 | ISP1820 celD::Tn10 (pSIF519) | This study |
| TRASH61 | FIM-76 | ISP1820 *waaK*::Tn10 (pSIF519) | This study |
| TRASH72 | *nuoH*::Tn10 pRS*fimA* | ISP1820 *nuoH*::Tn10 (pSIF519) | This study |
| DEF1697 | Δ*arcA* pRS*fimA* | ISP1820 Δ*arcA* (pSIF474) | This study |
| DEF1817 | Δ*barA* pRS*fimA* | ISP1820 Δ*barA* (pSIF474) | This study |
| DEF1445 | Δ*cpxR* pRS*fimA* | ISP1820 *ΔcpxR*(pSIF474) | This study |
| DEF1352 | Δ*crp* pRS*fimA* | ISP1820 Δ*crp* (pSIF474) | This study |
| DEF1348 | Δ*fimW* pRS*fimA* | ISP1820 *ΔfimW*(pSIF474) | This study |
| DEF1346 | Δ*fimY* pRS*fimA* | ISP1820 Δ*fimY* (pSIF474) | This study |
| DEF1345 | Δ*fimZ* pRS*fimA* | ISP1820 Δ*fimZ* (pSIF474) | This study |
| DEF1816 | Δ*flhCD* pRS*fimA* | ISP1820 Δ*flhCD*(pSIF474) | This study |
| DEF1349 | Δ*fliZ* pRS*fimA* | ISP1820 Δ*fliZ* (pSIF474) | This study |
| DEF1365 | Δ*seqA* pRS*fimA* | ISP1820 Δ*seqA* (pSIF474) | This study |
| DEF1677 | Δ*lrhA* pRS*fimA* | ISP1820 *ΔlrhA*(pSIF474) | This study |
| DEF1351 | Δ*lrp* pRS*fimA* | ISP1820 Δ*lrp* (pSIF474) | This study |
| DEF1353 | Δ*nagC* pRS*fimA* | ISP1820 Δ*nagC*(pSIF474) | This study |
| DEF1814 | Δ*narP* pRS*fimA* | ISP1820 Δ*narP* (pSIF474) | This study |
| DEF1473 | Δ*ompRΔenvZ* pRS*fimA* | ISP1820 *ΔompRΔenvZ* (pSIF474) | This study |
| DEF1530 | Δ*oxyR* pRS*fimA* | ISP1820 Δ*oxyR* (pSIF474) | This study |
| DEF2219 | Δ*phoP* pRS*fimA* | ISP1820 Δ*phoP* (pSIF474) | This study |
| DEF1823 | Δ*rcsB* pRS*fimA* | ISP1820 Δ*rcsB* (pSIF474) | This study |
| DEF1608 | Δ*rpoN* pRS*fimA* | ISP1820 Δ*rpoN* (pSIF474) | This study |
| DEF1824 | Δ*rpoS* pRS*fimA* | ISP1820 Δ*rpoS* (pSIF474) | This study |
| DEF1446 | Δ*sirA* pRS*fimA* | ISP1820 Δ*sirA* (pSIF474) | This study |
| DEF1724 | Δ*soxRS* pRS*fimA* | ISP1820 Δ*soxRS* (pSIF474) | This study |
| DEF1821 | Δ*tviA* pRS*fimA* | ISP1820 Δ*tviA* (pSIF474) | This study |
| DEF1350 | Δ*yqiC* pRS*fimA* | ISP1820 *ΔyqiC* (pSIF474) | This study |
| DEF1951 | *ndh*::Tn10 pRS*fimA* | ISP1820 *ndh*::Tn10(pSIF474) | This study |
| DEF2218 | *nuoH*::Tn10 pRS*fimA* | ISP1820 *nuoH* ::Tn10 (PSIF474) | This study |
| DEF2239 | Δ*oxyR-*C | ISP1820 Δ*oxyR* (pSIF650) | This study |
| ***S.* Typhimurium** |  |  |  |
| DEF1041 | WT | SL1344 wild-type | (Gulig et Curtiss, 1987) |
| DEF1549 | WT pRS | SL1344 (pRS415) | This study |
| DEF1954 | WT pRS*fimA* | SL1344 (pSIF474) | This study |
| DEF1521 | Δ*oxyR* | SL1344 Δ*oxyR* | This study |
| DEF1955 | Δ*oxyR*/pRS*fimA* | SL1344 Δ*oxyR* (pSIF474) | This study |
| ***E. coli*** |  |  |  |
| DEF1162 | MGN-617 | SM10 λpir *asd thi thr leu tonA lacY supE recA RP4 2-Tc : :Mu[λpir] asdA4* | (Kaniga et al., 1998) |
| DEF136 | BL21 (DE3) | F- *ompT hsdSB (rB-, mB-) gal dcm* (DE3) | (Studier et al.,1986) |
| **Plasmids** |  |  |  |
| pSIF149 | pMEG-375 | *sacRB mobRP4 oriR6K, Cmr Apr* | R. Curtiss III, U. Florida |
| pSIF117 | pRS415 | Multicopy vector with a promotorless, *lacZ* reporter gene, Ap | (Simons et al., 1987) |
| pSIF474 | pRS415 *fimA* | pRS415 carrying the promoter region of *fimA* | (Dufresne et al, 2018) |
| pSIF518 | pRS415 Cm | Multicopy vector with a promotorless, lacZ reporter gene, Cm | This study |
| pSIF519 | pRS415*fimA* Cm | pRS415 Cmr carrying the promoter region of *fimA* | This study |
| pSIF060 | pET14b | ori, T7 RNA polymerase, 6xHis-Tag, ApR | Novagen |
| pSIF650 | pET14b-oxyR | 6xHis-OxyR | This study |
| pSIF500 | pMEG-*fimW* | pMEG-375 with flanking region of *fimW* gene | This study |
| pSIF494 | pMEG-*fimY* | pMEG-375 with flanking region of *fimY* gene | This study |
| pSIF493 | pMEG-*fimZ* | pMEG-375 with flanking region of *fimZ* gene | This study |
| pSIF495 | pMEG-*fliZ* | pMEG-375 with flanking region of *fliZ* gene | This study |
| pSIF561 | pMEG-*rpoS* | pMEG-375 with flanking region of *rpoS* gene | This study |
| pSIF496 | pMEG-*yqiC* | pMEG-375 with flanking region of *yqiC* gene | This study |
