# Supplementary material for: Identification of OxyR as an activator of type 1 fimbriae (fim) in Salmonella enterica serovar Typhi
Source: Microbiol Spectr. 2025 Sep 15;13(10):e03267-24. doi: 10.1128/spectrum.03267-24 (PMC12502779; doi:10.1128/spectrum.03267-24)
Supplement: Table S2 — Table of primers. [file spectrum.03267-24-s0002.docx]

| **Table S2. Primers used in this study** | | |  |
| --- | --- | --- | --- |
| **Primers** | **Sequence (5'-3')*** | |  |
| RB1-RNA | CGGGATCCAGCTTCTCACGCANNNNNNNNN | | |
| RB1-PCR | CGGGATCCAGCTTCTCACGCA | | |
| pLOF F seq | CAAGACGTTTCCCGTTGAAT | | |
| STY:PCRNiche#2 | CTTGTGCAATGTAACATCAGAG | | |
| fimW F1 | CGGGATCCTCTCAGCACGCATAAAGTGG | | |
| fimW R2 | *TTTAACATG*GTCAATTTTCTGCTGCCAT | | |
| fimW F3 | *AAAATTGAC*CATGTTAAAACGGAGCAGCAGT | | |
| fimW R4 | GCTCTAGAGTGCCACCAAACACTCCTTC | | |
| fimY F1 | CGGGATCCGATGGAAGGCATTGAAACATCA | | |
| fimY R2 | *AGAAAGCTT*GAAATGATACCAACCGGCAAG | | |
| fimY F3 | *TATCATTTC*AAGCTTTCTTCGGCTGATCC | | |
| fimY R4 | GCTCTAGAAGATGTTGACGCTGGAGA | | |
| fimZ F1 | CGGGATCCTCCACCGTGTGGTGTGTAG | | |
| fimZ R2 | *CAAGTTTAG*CAACAGGATAAGTGCGCAGAT | | |
| fimZ F3 | *ATCCTGTTG*CTAAACTTGGCCTTCACTC | | |
| fimZ R4 | GCTCTAGATGCTACCCTGAAATTCTATGCG | | |
| fliZ F1 | CGGGATCCTCAAACGATTACGCACCAA | | |
| fliZ R2 | *CGCAGTGCG*GTAATGCGGTCGAGCAATTT | | |
| fliZ F3 | *CCGCATTAC*CGCACTGCGTAAATACCA | | |
| fliZ R4 | GCTCTAGACGTCCCAGCAGTGCTAATTT | | |
| OxyR_F_NdeI | GGAATTCCATATGAATATTCGTGATCTTGAATATCTGGTG | | |
| OxyR_R_BamHI | CGGGATCCTTAAACCGCCTGTTTTAACGC | | |
| oxyR F1 | CGGGATCCTTATAAGCGTAGCGCCATCA | | |
| oxyR R2 | *CTCATAACG*CATAACGCCCAGCTCATCTT | | |
| oxyR F3 | *GGCGTTATG*CGTTATGAGCAACTGGCAGA | | |
| oxyR R4 | GCTCTAGAATCCCCACCGGGATTTATAC | | |
| rpoS F1 | cgggatccCCGGGAACAACAGGAAGTTA | | |
| rpoS R2 | *ACGTTCACG*CTCATCAAATTCCGCGTCTT | | |
| rpoS F3 | *TTTGATGAG*CGTGAACGTGTTCGTCAGAT | | |
| rpoS R4 | gctctagaCACTATCCACAAGCGTTTCG | | |
| yqiC F1 | CGGGATCCCCGTGATGAGAAATGCGTTA | | |
| yqiC R2 | *GCTTGTCGC*CGAACTCGCGAATACCTTT | | |
| yqiC F3 | *GCGAGTTCG*GCGACAAGCCTGAAGAAGTAA | | |
| yqiC R4 | GCTCTAGAGCAATATGGACGAGGAGCAC | | |
| FimA promF | FAM/CTTTACGTTTGCTTGCGACA | | |
| FimA prom R2 | CGGGATCCAGTAGGATCAGCCGCAACC | | |
| KatG F1 | CGGGATCCCTGCCGGGAGCTTTATTACA | | |
| KatG R2 | *GTTGGAACC*AAGATCCACACGAAGCTG | | |
| RecA F1 | AGAAGATTGGCCAGGGTAAAGCGA | | |
| RecA R2 | GAA ATC GGG CGT GGC ATT CTG ATT | | |
| *Restriction enzyme sites are underlined. Letter in italics represent overlapping sequences. | | | |
|  |  |  |  |
|  |  |  |  |
|  |  |  |  |
|  |  |  |  |
|  |  |  |  |
|  |  |  |  |
